# Editorial: Hearing loss: from pathogenesis to treatment, volume II

**DOI:** 10.3389/fncel.2023.1309592

**Published:** 2023-10-23

**Authors:** Shengyu Zou, Qingyin Zheng, Yu Sun, Xiaolong Fu, Wenjie Zhou, Zuhong He

**Affiliations:** ^1^Department of Otorhinolaryngology-Head and Neck Surgery, Zhongnan Hospital of Wuhan University, Wuhan, China; ^2^Department of Otolaryngology-Head and Neck Surgery, Case Western Reserve University, Cleveland, OH, United States; ^3^Department of Otorhinolaryngology, Union Hospital, Tongji Medical College, Huazhong University of Science and Technology, Wuhan, China; ^4^Shandong Provincial Hospital, Shandong First Medical University, Jinan, China; ^5^Songjiang Research Institute and Songjiang Hospital, Shanghai Jiao Tong University School of Medicine, Shanghai, China

**Keywords:** hearing loss, presbycusis, signaling pathways, epigenetic, hair cell regeneration

Hearing impairment is an important disabling disease worldwide. However, there is still no effective method for damaged auditory hair cell regeneration. This Research Topic contains basic and clinical research and a review of manuscripts related to the pathogenesis and intervention of hearing diseases.

To restore hearing function in those with severe hearing loss, cochlear implantation (CI) is a well-established surgical treatment. However, some patients may experience residual hearing loss after undergoing CI surgery. Ernst et al. supposed there are two key concepts with residual hearing preservation. First, the surgeon must follow with soft surgery and administer corticosteroids to the patient after surgery either systemically or intratympanically. Second, the appropriate CI electrode array must be determined by the cochlear anatomy and pattern of hearing loss of each patient. Their group further investigated inducible NO synthase isoform (iNOS) expression in different cell types of the inner ear after CI surgery. Through the detection of physiological indicators in the cochlea, it was found that compared with patients in the sham surgery group, post-CI patients experienced decreased cochlear blood flow after surgery, and the cochlear microvascular permeability substantially increased. The iNOS expression levels decreased in many cell types in the organ of Corti after CI surgery. Therefore, the author suggests that the disturbance of the inner ear microenvironment after CI surgery may be related to changes in NO-related signaling pathways. Inhalative application of NO or pharmacological induction of iNOS may provide a therapeutic regimen for residual hearing preservation during CI surgery.

Ma et al. focused on the role of miR-29a during aging-related hearing loss. By evaluating the hearing level, they found that miR-29a mutant mice exhibited poor hearing levels compared with littermates at 2 months after birth, and those mice had hearing loss that gradually worsened until 5 months later. Subsequently, they discovered cochlear pathology changes in *miR-29a*^−/−^ mice and showed that the stria vascularis was thinner and that spiral ganglion neurons were decreased in *miR-29a*^−/−^ mice. Out hair cells (OHCs) and inner hair cells (IHCs) were significantly lost, and stereocilia fusion was observed in miR-29a KO mice. GO annotation and KEGG pathway enrichment analysis revealed that the miR-29 family is related to extracellular matrix gene expression. Col4a family gene and laminin family gene expression was increased after miR-29a deficiency and could induce basal membrane thickening. These results elucidate new mechanisms for the onset of age-related hearing loss and provide new ideas for presbycusis treatment.

As a canonical antitumor drug, cisplatin has been widely used in the clinic for malignant tumor treatment. However, cisplatin has greatly limited use due to ototoxicity and neurotoxicity. Li et al. reviews cisplatin ototoxic mechanisms and prevention strategies. In addition to the apoptotic pathway, the author also introduced the mechanisms of autophagy and mitophagy in cisplatin ototoxicity. More evidence has shown that apoptosis and the mitophagy pathway have the same regulator, and foreign drugs can induce the activation of the mitophagy pathway to antagonize cisplatin ototoxicity. The author also provides the most recent approaches to cisplatin ototoxicity prevention and treatment in this review, including some new drugs currently undergoing clinical trials. In terms of drug research and development, the main focus is on mechanisms such as antioxidant activity, inhibition of transporter proteins, and regulation of apoptosis and autophagy signaling pathways. The recent development of materials science has also provided new methods for the prevention and treatment of cisplatin ototoxicity. The agents can easily pass through the inner ear blood-labyrinthic barrier and provide new technologies for drug delivery.

Noise-induced hearing loss (NIHL) is a major public issue causing hearing impairment. Lai et al. has performed much work on NIHL and explored the important role of calcium channels in NIHL. Recent research results from Lai indicate that MDL-28170 could inhibit calpain activation, which is a family of calcium-dependent cysteine proteases. Intraperitoneal injection of MDL-28170 before noise exposure attenuated noise-induced hearing loss and cochlear pathologies. As a substrate for calpain-1, its activation during noise exposure could be inhibited by MDL-28170. Additionally, MDL-28170 therapy increased p85 and p-Akt (s473) in OHCs and reversed the decrease in PI3K/Akt signaling following noise exposure. These results show that treatment with MDL-28170 is a promising strategy for NIHL prevention.

As the most common gene mutation that could cause congenital non-syndromic deafness, the *GJB2* gene plays an important role in cochlear development and maintaining HC normal function. Wang et al. reviewed GJB2-related inner ear disorders and hearing loss. They suggested that *Gjb2* mutation caused K^+^ circulation impairment may not be the main pathological mechanism of deafness. They also introduced the *Gjb2* gene mutation that caused cochlear structure development disorder and mainly focused on the tunnel of Corti and Nuel's space formation. Furthermore, the manuscript introduced the idea that *Gjb2* mutation will cause cochlear sensory epithelium energy supply deficiency. Therefore, cochlear nutritional deficiency causes an increase in oxidative metabolites and affects antioxidant defense pathway activation. Wang et al. further proposed that *Gjb2* mutation-induced Cx26 dysfunction can affect inner ear ATP release and Ca^2+^ signaling transmission, which can reduce active cochlear mechanics and hearing sensitivity.

Epigenetic regulation plays an important role in gene transcription and regulation. Xiao and Li reviewed epigenetic regulation in HC survival and regeneration. They introduced epigenetic regulation in the cell cycle and inner ear HC development. Epigenetic regulation will affect HC formation signaling and spiral ganglion neuron development pathways. Furthermore, epigenetic modification plays a critical role in D-gal-induced mimicking of presbycusis, ototoxic drug-induced hearing loss and noise-induced hearing loss. Researchers have also used compounds to regulate epigenetic modification to alleviate acquired hearing loss. Epigenetic regulation is also crucial for inner ear HC regeneration, which offers an opportunity for hearing loss therapy. By regulating DNA epigenetics, inner ear progenitor cells will change their proliferation and transdifferentiated abilities and further induce progenitor cell lines into hair cell-like cells.

As a marker of tissue-resident stem cells, LGR5 is expressed in mammalian cochlear supporting cells (SCs), and LGR5^+^ SCs are regarded as endogenous progenitor cells for HC regeneration. Smith-Cortinez et al. tracking of LGR5 expression in mature adult mice between P30 and P200 showed that Lgr5 mRNA expression was not significantly different between Lgr5^GFP^ mice and WT mice. Furthermore, they used furosemide plus kanamycin injection to induce hair cell damage and found that Lgr5 expression persisted 28 days after ototoxic damage in SCs. Through mRNA level validation, they found that the expression of Lgr5 increased after 7 days of acute ototoxic injury and returned to normal levels after 28 days. These results provide a therapeutic window for inner hair cell regenerative therapies in the future.

For this Research Topics include cochlear implantation, the pathogenesis of presbycusis, the prevention of noise-induced hearing loss and inner ear hair cell regeneration-related research. These research findings provide new ideas for the prevention and treatment of acquired hearing loss. Furthermore, they also provide evidence for the time window of sensory hair cell regeneration after injury.

## Author contributions

ZH: Supervision, Validation, Writing—original draft, Writing—review and editing. SZ: Writing—original draft. QZ: Writing—review and editing. YS: Writing—review and editing. XF: Writing—review and editing. WZ: Writing—review and editing.

